# Enhanced uptake of gH625 by blood brain barrier compared to liver *in vivo*: characterization of the mechanism by an *in vitro* model and implications for delivery

**DOI:** 10.1038/s41598-018-32095-w

**Published:** 2018-09-14

**Authors:** Annarita Falanga, Giuseppina Iachetta, Lucia Lombardi, Emiliana Perillo, Assunta Lombardi, Giancarlo Morelli, Salvatore Valiante, Stefania Galdiero

**Affiliations:** 1 Department of Pharmacy, Via Mezzocannone 16, 80134 Napoli, Italy; 2 CiRPEB- University of Naples “Federico II”, Via Mezzocannone 16, 80134 Napoli, Italy; 3 Department of Biology, University of Naples “Federico II”, Via Mezzocannone 8, 80134 Napoli, Italy; 4 National Institute of Biostructures and Biosystems (INBB), V. le Medaglie d’Oro, 00136 Rome, Italy

## Abstract

We have investigated the crossing of the blood brain barrier (BBB) by the peptide gH625 and compared to the uptake by liver *in vivo*. We clearly observed that *in vivo* administration of gH625 allows the crossing of the BBB, although part of the peptide is sequestered by the liver. Furthermore, we used a combination of biophysical techniques to gain insight into the mechanism of interaction with model membranes mimicking the BBB and the liver. We observed a stronger interaction for membranes mimicking the BBB where gH625 clearly undergoes a change in secondary structure, indicating the key role of the structural change in the uptake mechanism. We report model studies on liposomes which can be exploited for the optimization of delivery tools.

## Introduction

Central nervous system (CNS) diseases constitute the largest area of unmet health demand; in fact, despite the presence of candidate therapeutic molecules, drug delivery is made difficult by the presence of the blood-brain barrier (BBB), which ultimately results in the limited entry of 95% of drug candidates which may be valuable for CNS diseases. The BBB is made by brain capillary endothelial cells closely interconnected with tight junctions which effectively seal the capillary wall eliminating any inter-endothelial space. In addition, there are efflux transporters which also play a key role in obstructing the entrance into the CNS^1,2^. Thus, crossing the BBB is a major challenge which has also been addressed by the use of nanosystems; although nanosystems may present several advantages for drug delivery in general, still the amount of drug that enters the brain is very low^3,4^. A key strategy to enhance brain delivery is represented by surface modification with ligand binding moieties or receptors expressed at the luminal surface of cerebral endothelial cells^5^. Two main processes have been put forward to elucidate the transport mechanism: receptor-mediated transcytosis (RMT) and adsorptive-mediated transcytosis (AMT)^6,7^.

Cell-penetrating peptides (CPPs) are short amphipathic and positively charged peptides acting as cargo carriers which enter complex physiological barriers without cell rupture thus in a non-invasive manner and without the aid of cell membrane receptors^8^. Although, evidences of the exact internalization mechanism are scarce and elusive, it has been commonly accepted that more than one mechanism may be involved^7^. Considering the major endocytic entry pathway, the general application of CPPs in drug delivery has been complicated by their entrapment in cellular organelles, which seriously reduces the cargo delivering efficacy.

Moreover, a current hotspot in CPP research is to understand whether their ability to cross the biological membranes also implies their ability to pass the BBB^9^. Although positively charged CPPs would be proper candidates to assist brain drug delivery via the AMT mechanism, scarce literature reports actually show their capacity to deliver cargoes through the BBB and often they result in divergent outcomes. As a matter of fact, a detailed overview with comparable, quantitative data of the currently available brain influx studies of CPPs is unavailable. Some authors showed that CPPs are able to reach the brain parenchyma both *in vivo* and *in vitro*^10^. *In vivo* evaluation of some CPP resulted in compromised BBB; as a matter of fact, although Tat-mediated delivery of neuroprotective drugs across the BBB in ischemia and seizure models were very promising, it was evidenced that the BBB was compromised^9,11^.

AMT delivery is triggered by electrostatic interactions between positive charges on the nanosystem and relative high concentration of negative charges on the membrane of brain endothelial cells^7^; however, positively charged particles often exhibit a rapid blood clearance together with aggregation problems and high accumulation in the lung and liver^12^. Therefore, the number and type of charges present on the surface of the nanosystem play a key role in CNS drug delivery.

In particular, the tissue distribution varies among the different CPPs, with most of them showing a higher liver distribution compared to other tissues, which can indicate metabolization, or uptake of the peptides and of their metabolites into hepatocytes^9,13^.

Considering that positively charged molecules may form aggregates in the presence of negatively charged serum proteins and result in enhanced accumulation in the lung and liver, less positive charged CPP may represent an alternative in order to achieve a more effective brain delivery. Recent progresses in the use of membranotropic peptides for the delivery of macromolecules across cell membranes, provide a great opportunity for CNS delivery^14^. These amphipathic peptides are capable of causing the membrane distortion necessary for insertion into the target membrane. Recently, the peptide gH625, belonging to this class of membranotropic sequences, showed remarkable vector properties, efficiently delivering a range of cargoes^15^. gH625 is essentially internalized by a non endocytic mechanism avoiding endosomal entrapment^16–23^ and is also shown to be able to cross the BBB *in vitro* and *in vivo*^16,24^. The peculiar ability of crossing physical barriers is probably correlated to the amino acid residues which seem to be central for the interaction and destabilization of the target lipid membranes; moreover, it is able to assume an amphipathic α-helical structure in membranes which is key to favouring the binding of proteins to membranes^25^. A mutagenesis study was done previously to understand the role of each residue in the peptide-lipid interaction, fusion and helix formation^25–28^.

In a previous work, gH625 was shown to be able to cross the BBB *in vivo*^24^. Uptake was not linked to alterations of cell viability or maximal mitochondrial oxidative capacity, suggesting that gH625 could potentially be used as a brain delivery system for macromolecules^24^. In general a high accumulation in the target tissue results in an improved therapeutic effect, while a large amount of drug distributed to non-target organs may cause unwanted toxicity and/or side effects. It is thus fundamental to reduce liver metabolism and renal clearance to produce prolonged blood circulation with an increased chance of accumulation in the target tissue^12^. Therefore, in order to employ gH625 as an attractive CPP for facilitating CNS delivery, it is fundamental to understand the affinity of the peptide for the BBB and for the liver. We started our analysis from *in vivo* bio-distribution analysis between BBB and liver and then using a combination of techniques and model membranes, we gained further insight into the mechanism of internalization which will help in the optimization of this and similar delivery tools.

## Experimental Section

### Materials

1-palmitoyl-2-oleoyl-*sn*-glycero-3-phosphocholine (POPC), dioleoyl phosphatidylcholine (DOPC), dioleoyl phosphatidylglycerol (DOPG), Sphingomyelin (SM), N-(7-nitro-benz-2-oxa-1,3-diazol-4-yl) phosphatidylethanolamine (NBD-PE) and N-(Lissamine-rhodamine-B-sulfonyl) phosphatidylethanolamine (Rho-PE) were purchased from Avanti Polar Lipids (Birmingham, AL, USA), while cholesterol (CHOL), Triton 100X and 4-chloro-7-nitrobenz-2-oxa-1,3-diazole (NBD-Cl) were purchased from Sigma (St. Louis, MO, USA). DAPI (4′,6-Diamidino-2-Phenylindole, Dilactate) was purchased from Invitrogen (Carlsbad, CA, USA), Sudan Black B from Sigma-Aldrich (St. Louis, MO, USA). Picric acid, acetic acid, formaldehyde and paraffin wax were from Carlo Erba (Milan, IT).

### Peptide synthesis

Synthesis of the peptide gH625 (NH_2_-HGLASTLTRWAHYNALIRAF-CONH_2_) was performed via standard solid-phase-9-fluorenylmethoxycarbonyl (Fmoc) chemistry as previously reported^29^. The peptide was obtained with a yield of 30–40% and purity was higher than 98%. Labelling of the N-terminus with 4-chloro-7-nitrobenz-2-oxa-1,3-diazole (NBD-Cl) to obtain NBD-gH625 was performed in N,N-Dimethylformamide (DMF) with 2 equivalents of N,N-Diisopropylethylamine (DIPEA) as previously reported^23^.

### Animals

*In vivo* studies were performed in male Wistar rats housed under a 12 hours light-dark cycle with free access to food and water. The animals were allocated in five groups, each consisting of three rats. Three animal groups received a single i.v. administration of NBD-gH625 at a concentration of 10 µg, 20 µg, and 40 µg/100 g bw, respectively. Control group received vehicle while another group received gH-625 (40 µg/100 g bw). Both NBD-gH625 and gH625 were dissolved in phosphate-buffered saline. After 3.5 hours from the i.v. administration, the animals were anesthetized with Zoletil® (40 mg/100 g) and euthanized by decapitation. This study was carried out in strict accordance with recommendations in the European Guide for the Care and Use of Laboratory Animals. Every effort was made to minimize animal pain and suffering. All animal protocols were approved by the Committee on the Ethics of Animal Experiments of the University of Napoli Federico II (Italy) and the Italian Minister of Health. (Protocol number 2012/000180).

### Fluorescence microscopy analysis

The *ex-vivo* fluorescence microscopy analysis was performed to evaluate the distribution of NBD-gH625 on both sides of the BBB. In order to evaluate if there is a linear relationship between slides fluorescence integrated density and the amount of the peptide, a calibration curve was obtained. In particular, increasing amounts of NBD-gH625 (0–30 µM) were spotted on microscope slides and images at 515 nm were acquired (See the Results section) and analysed as described below. Brains were removed from animals, sectioned sagittally in thick slices, washed in PBS, mounted on coverslips, stained with DAPI (4′–6 Diamidino-2-Phenylindole, Dilactate) (Invitrogen, Carlsbad, CA, USA) and analysed under Axioskop fluorescence microscope (Zeiss). Images, at least 10 field of view (800 × 700 μm each) for each experimental class, were acquired using an AxioCam MRc5 (Zeiss) with DAPI filter (λ_ex_ 350 nm; λ_em_ 461 nm) and a fluorescein isothiocyanate filter (λ_ex_ 488 nm; λ_em_ 515 nm).

To evaluate the distribution of NBD-gH625 on both sides of the BBB, we estimated the fluorescence inside the blood vessels (without discriminating between the amount present in the blood and that present in the endothelial cells) and outside the blood vessels in 10 images for each experimental class. In detail, to assess the NBD-gH625 that reached without crossing the BBB, we selected blood vessels as the region of interest (ROI) (inbound) and evaluated the fluorescence intensity; then, on the non-zero thresholded images, an inversion of selected ROI was carried out to obtain the complementary ROI to calculate the amounts of NBD-gH625 which crossed the BBB (outbound).

The morphology of blood vessels was easily identified and outlined; the images were calibrated by ImageJ Rodbard function; integrated density values were calculated within the outlines exclusively, to be considered as inside fluorescence. Then, the thresholded and outlined images, to exclude both blood vessels and background values, were used to calculate the integrated density as outside fluorescence.

To evaluate the effect of concentration on the systemic delivery of NBD-gH625, a fluorescence microscopy analysis was performed as previously reported^24^.

Briefly, livers and brains were isolated and fixed for 24 hours in Bouin’s fixative (71% picric acid, 5% acetic acid, 24% formaldehyde) and embedded in paraffin wax (Carlo Erba, Milan, Italy). Tissue sections (10 μm of thickness) were incubated in 0.1% Sudan Black B dissolved in 70% ethanol for 20 minutes to reduce the tissues autofluorescence. The excess of Sudan Black B was removed by washing with Tris-buffered saline containing 0.02% Tween 20^30,31^. Sections were stained with DAPI to counterstain nuclei, then mounted with a coverslip in glycerol/phosphate-buffered saline (1:1) and acquired using an AxioCam MRc5 with DAPI filter (λ_ex_ 350 nm; λ_em_ 461 nm) and a fluorescein isothiocyanate filter (λ_ex_ 488 nm; λ_em_ 515 nm). All images were then analysed using Fiji 1.50 software to determine the mean fluorescence and the integrated density, defined as the product of the mean fluorescence per the region of interest (μm^2^)^32^. The percentage of delivered vs administered NBD-gH625 was calculated.

### Statistical analysis

Statistical analysis was performed using GraphPad Prism 5.0. The two-tailed Mann Whitney t-test was used and P was considered significant if was at least less than 0.05.

### Liposome preparation

Large unilamellar vesicles (LUVs) and small unilamellar vesicles (SUVs) consisting of PI/DOPE/DPPC/PS/Chol/SM (4/23/30/8/20/15 ratio in moles)^33,34^, DSPC/POPE/PI/PS/lysoPC/SM/DOPG (37/21/7/9/3/18/5 ratio in moles)^35^, and when required containing Rho-PE and NBD-PE, were formulated in 5 mM HEPES, 100 mM NaCl, pH 7.4. LUVs were prepared using the extrusion method as previously reported^36^; lipids were first dissolved in chloroform, then dried under a nitrogen gas stream and lyophilized overnight. For fluorescence experiments, buffer was added to dry lipid films and vortexed for 1 h; then the lipid suspension was freeze-thawed 6 times and extruded 20 times through polycarbonate membranes with 0.1 μm diameter pores to obtain LUVs. For circular dichroism measurements, peptide samples in SUVs were prepared using the following protocol^37^. Lipids were dissolved in chloroform and added to an equal volume of peptide solution dissolved in TFE containing appropriate peptide concentration. The samples were vortexed and lyophilized overnight. The dry samples were rehydrated with phosphate buffer (5 mM) for 1 h and sonicated for 30 min.

### Membrane fluidity

LUVs containing Laurdan were prepared to determine membrane fluidity^23^. In order to insert Laurdan into the bilayer, it was added to lipid films before hydration. The peptide gH625 was added to LUVs at a P/L molar ratio of 0.5 and after 10 min the fluorescence spectra were recorded using a 1 cm path length quartz cell, thermostated at 25 °C or 37 °C. Spectra were corrected for the baseline signal. Laurdan emission spectra were recorded from 400 to 550 nm with λ_ex_ 365 nm in the absence or presence of gH625 in 5 mM HEPES, 100 mM NaCl buffer (pH 7.4). Laurdan emission can shift from 440, in the ordered phase, to 490 in the disordered phase. The excitation generalized polarization (GP) was calculated as GP = (I_440_ − I_490_)/(I_440_ + I_490_) where I_440_ and I_490_ are the fluorescence intensities at the maximum emission wavelength in the ordered (λ_em_ 440 nm) and disordered (λ_em_ 490 nm) phases^38^.

### Lipid mixing assays

Membrane lipid mixing was followed by the resonance energy transfer assay (FRET)^39^. The peptide was added to vesicles containing 0.6 mol % of two probes, NBD-PE (donor) and Rho-PE (acceptor), mixed with unlabelled vesicles at a 1:4 ratio (final lipid concentration 0.1 mM). The dilution of NBD-PE and Rho-PE due to membrane mixing produced an increase in NBD fluorescence (λ_ex_ 465 nm, λ_em_ 530 nm) that was monitored at 37 °C as a function of added peptide. In order to avoid scattering interferences, a cut off filter (515 nm) between the sample and the emission monochromator was used. Complete mixing (100% level) was evaluated as the fluorescence intensity of vesicles after the addition of Triton X-100 (0.05% v/v) at the same total lipid concentrations of the fusion assay, while the zero level corresponded to the initial residual fluorescence of the vesicles. In order to get reproducible data and average results, lipid mixing experiments were repeated at least three times.

A modification of the lipid mixing experiment allowed to measure peptide-induced mixing of the inner monolayer^40^. A concentration of 0.6% mol for each of the fluorescent probes within the liposome was used. In order to completely reduce the NBD located at the outer surface of the membrane, LUVs were treated with sodium dithionite 100 mM (from a stock solution of 1 M dithionite in 1 M TRIS, pH 10.0) for approximately 1 h on ice in the dark. Excess sodium dithionite was removed by size exclusion chromatography through a Sephadex G-75 50 DNA Grade filtration column (GE Healthcare) eluted with a buffer containing 10 mM TRIS, 100 mM NaCl, and 1 mM EDTA, pH 7.4.

The ANTS/DPX experiment was used to evaluate leakage of encapsulated dyes induced by the peptide. The peptide in 5 mM Hepes and 100 mM NaCl at pH 7.4 was added to the stirred vesicle suspension (0.1 mM lipid) containing ANTS and DPX at 37 °C. Details of this assay have been previously reported^41^.

### Tryptophan fluorescence measurements

Emission spectra of the tryptophan contained in the peptide (4 µM) in the absence or presence of target vesicles were recorded between 300 and 400 nm with λ_ex_ 295 nm in order to determine the presence of the peptide inside the membrane. To establish the strength of peptide-lipid interaction, LUVs were added to the peptide solution (4 µM) and the fluorescence intensity was recorded as a function of the lipid/peptide molar ratio (L/P), in three to four separate experiments. The results were corrected for the dilution factor. We used an L/P ratio of 200:1. The obtained binding was described as a partition equilibrium: X_b_ = K_p_C_f_ where K_p_ is the apparent partition coefficient in units of M^−1^, X_b_ is the molar ratio of bound peptide per total lipid and C_f_ is the equilibrium concentration of the free peptide in solution^42^. F_∞_ was obtained by extrapolation of a double reciprocal plot of the total peptide fluorescence vs the total lipid concentration in the outer leaflet. The fraction of membrane-bound peptide, fb, was determined by f_b_ = (F − F_0_)/(F_∞_ − F_0_), where F represents the fluorescence of peptide after the addition of the vesicles and F_0_ represents the fluorescence of the unbound peptide. f_b_ allowed us to calculate the equilibrium concentration of the free peptide in the solution, C_f_, and the extent of peptide binding X_b_. Values of X_b_ were corrected as X_b_^*^ = X_b_/0.6, considering that the peptides were initially partitioned only over the outer leaflet of the SUV (60% the total lipid). The binding isotherm is the curve resulting from plotting X_b_^*^ versus the concentration of the free peptide, C_f_.

### Tryptophan quenching by acrylamide

A solution containing 4 µM of gH625 in the absence or presence of LUVs at a peptide/lipid molar ratio of 1/200, was titrated with the water-soluble quencher acrylamide (4 M mother solution). The maximal concentration of acrylamide in the samples is 0.2 M. Fluorescence was measured with λ_ex_ 295 nm and λ_em_ 340 nm^43^. The data were analysed with the Stern-Volmer equation^43^, F_0_/F = 1 + Ksv [Q], where F_0_ and F represent the fluorescence intensities in the absence and the presence of the quencher (Q), respectively. The K_sv_ is the Stern-Volmer quenching constant, and is correlated to the accessibility of the tryptophan residue to acrylamide. As acrylamide does not significantly partition into the membrane bilayer, K_sv_ is a reliable reflection of the bimolecular rate constant for collisional quenching of the aromatic residues present in the aqueous phase^43^. K_sv_ is thus determined by the amount of non-lipid-associated free peptide as well as the peptide fraction located on the surface of the bilayer.

### Circular dichroism spectroscopy

CD spectra were recorded at room temperature on a Jasco J-715 spectropolarimeter in a 0.1 cm quartz cell. The spectra are an average of 3 consecutive scans from 260 to 190 nm, recorded with a band width of 3 nm, a time constant of 16 s, and a scan rate of 10 nm/min. Spectra were recorded and corrected for the blank. Mean residue ellipticities (MRE) were calculated as MRE = Obsd/lcn (Obsd is the ellipticity measured in millidegrees, l is the path length of the cell in cm, c is the peptide concentration in mol/l, and n is the number of amino acid residues in the peptide). A solution of 8 µM of peptide with SUVs, was prepared as previously described^37^. The measurements were performed at P/L ratio of 0.08 mol/mol in SUVs consisting of PI/DOPE/DPPC/PS/Chol/SM (4/23/30/8/20/15 ratio in moles) and DSPC/POPE/PI/PS/lysoPC/SM/DOPG (37/21/7/9/3/18/5 ratio in moles). The percentage of helix was calculated from measurements of their mean residue ellipticity at 222 nm^44^. We used [ϑ]_222_ values of 0 and −40.000(1–2.5/n) deg cm^2^ dmol^−1^ per amino acid residue for 0% and 100% helicity; n is the number of amino acid residues. The ratio of the ellipticities at 222 and 208 nm were used to discriminate between monomeric and oligomeric states of helices^45^. The experiment was performed before and after centrifugation to remove eventual precipitated peptides from solution.

## Results

### Fluorescence microscopy analysis of rat tissues

**Figure 1:**
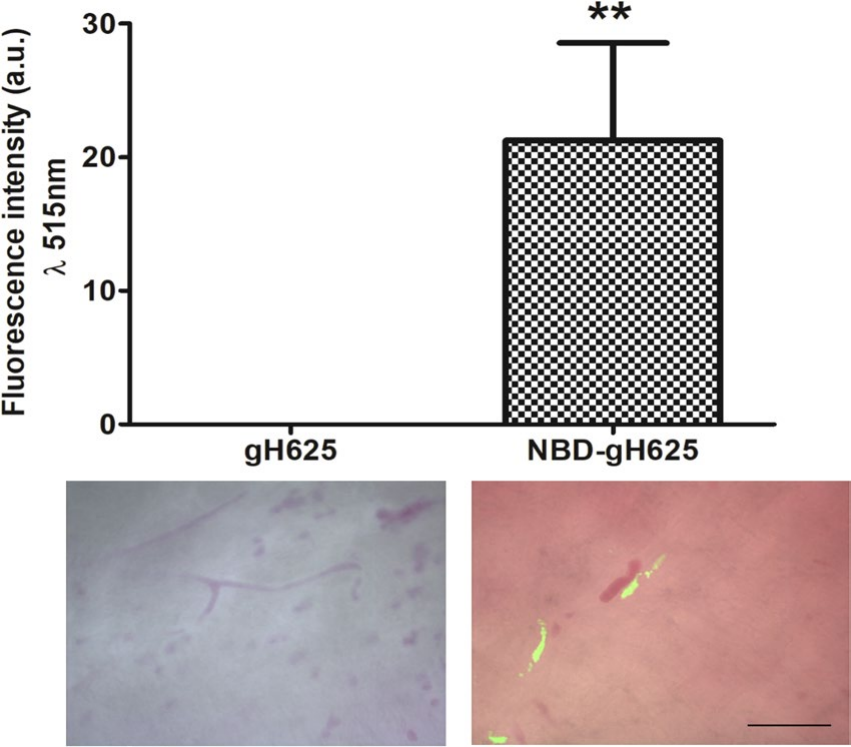
Evaluation of the fluorescence signal on *ex vivo* brain samples at 515 nm. Images show no signal in gH625 samples and intense fluorescence in NBD-gH625 samples. Fluorescence appears to be inside body cells. Scale bar 50 μm. **P < 0.01.

To evaluate the amount of green fluorescence related to the peptide in brain, we compared the fluorescence of gH625 and NBD-gH625 treated tissue samples. To evaluate the suitability of this approach we used as internal control the DAPI fluorescence and found the same amount of fluorescence at 461 nm for both gH625 and NBD-gH625 (Supplementary Material S1). When we evaluated the fluorescence at 515 nm we found no emission for gH625, thus in our study samples from animals administered with gH625 were used as “blank”; conversely, we measured a consistent increase of fluorescence in NBD-gH625 samples (Fig. 1) being mostly of the cell-specific.

**Figure 2:**
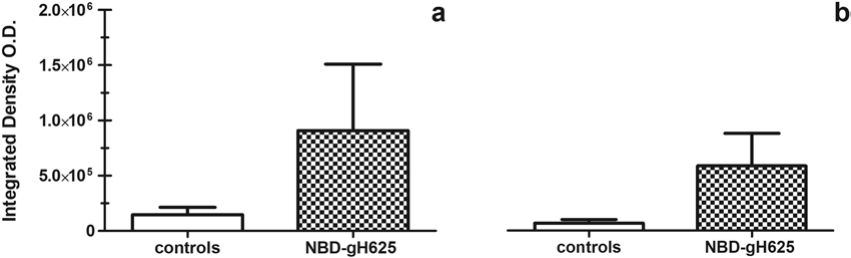
*In vivo* brain uptake of NBD-gH625 measured by integrated density values of fluorescence. (**a**) NBD-gH625 delivered to the BBB. (**b**) NBD-gH625 crossing the BBB and found in the brain parenchyma.

We thus used NBD-gH625 for all our analysis. The analysis of fluorescence values inside and outside blood brain vessels showed that NDB-gH625 reaches the BBB of treated animals (Fig. 2a) and/or crosses the BBB (Fig. 2b).

**Figure 3:**
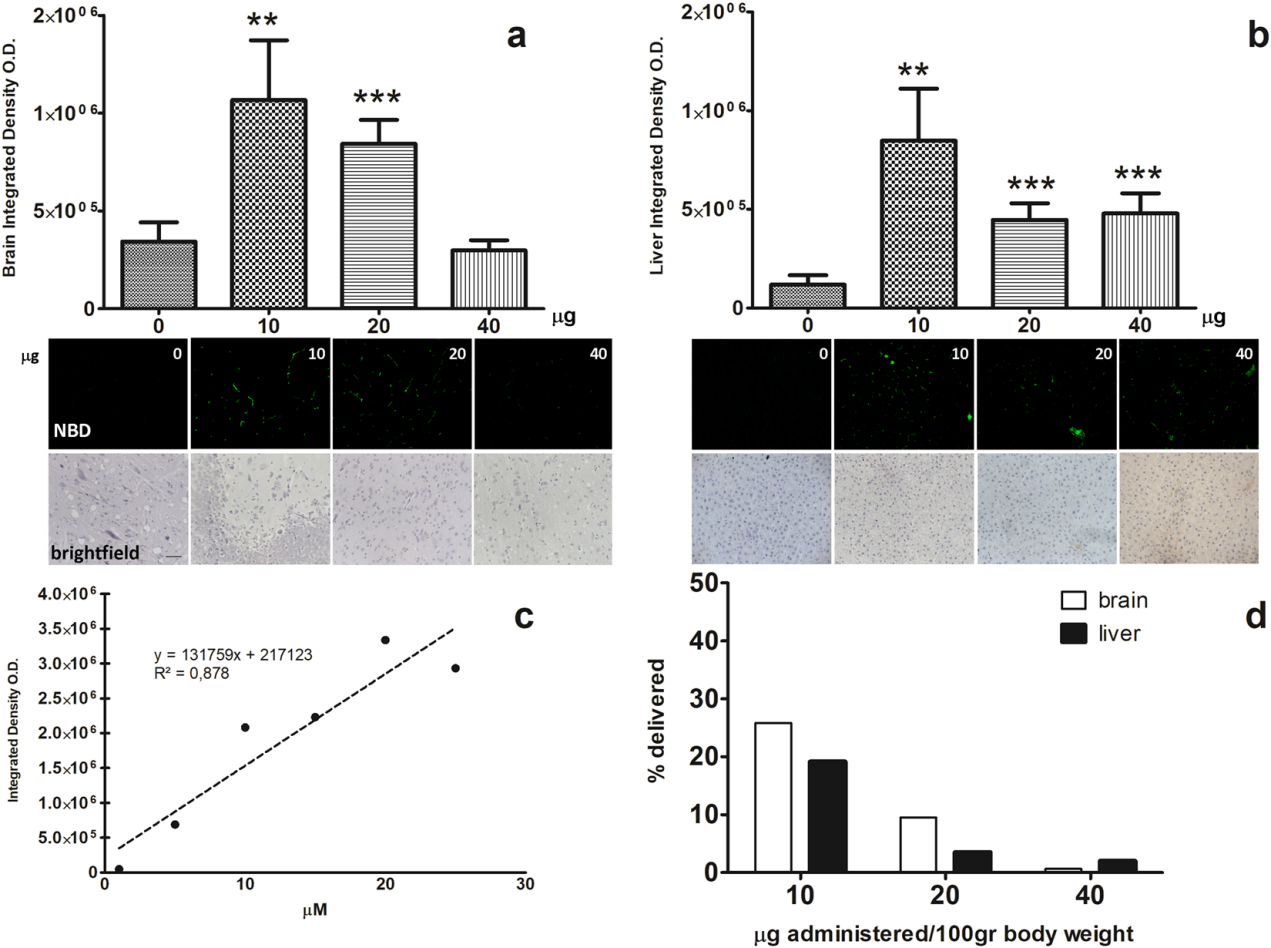
Fluorescence microscopy analysis of brain and liver slices of NBD-gH625 treatment. Experimental classes are expressed in μg per 100 gr of body weight. Scale bar 50 μm. (**a**) Integrated density of brain slices from rats injected with different concentration of NBD-gH625. **P < 0.01, ***P < 0.001. (**b**) Integrated density of liver slices from rats injected with different concentrations of NBD-gH625. **P < 0.01, ***P < 0.001. (**c**) Calibration curve representing the linear relationship between the known amounts of NBD-gH625 and the relative the integrated density obtained under the setup of the microscopy analysis system used. (**d**) Percentage of NBD-gH625 delivered in brain and liver after *in vivo* administration of NBD-gH625.

To evaluate the extent of this NBD-gH625 withdrawal from blood by liver sequestration, we compared different concentrations of NBD-gH625 injected in rats. Our data concerning fluorescence levels on brain slices, suggest that lower peptide concentrations are able to improve the reaching of the brain more compared to higher concentrations (Fig. 3a) as demonstrated by the highest concentration administered (40 μg) which produced an effect comparable to the control.

In the liver, we found that the lowest dose of NBD-gH625 has higher effect, giving rise to an increase of integrated density values compared to the higher ones, thus concentrating the fluorescence signal in narrow area (Fig. 3b); it remains to be determined whether this is due to a more effective removal of the peptide by Kupffer cells or a diffuse endothelial uptake as reported in literature^46^.

We also estimated the percentage of NBD-gH625 delivered in brain and liver, as the integrated density calibrated function of known amounts of NBD-gH625 (Fig. 3c). We showed that lower doses produced higher percentages of delivered peptide, while higher doses do not reach efficiently both organs (Fig. 3d).

### Membrane fluidity

LUVs fluidity before and after the addition of gH625 were obtained using the fluorescent probe Laurdan^47,48^. Laurdan is able to insert into membranes and distribute equally between lipid phases; it has an emission maximum at 440 nm in gel phase membranes and at 490 nm in liquid phase membranes. The Generalized Polarization (GP) is a parameter commonly used to quantify the change in the lipid fluidity. LUVs composed of PI/DOPE/DPPC/PS/Chol/SM (4/23/30/8/20/15) mimicking the composition of the BBB^34^ and of DSPC/POPE/PI/PS/lysoPC/SM/DOPG (37/21/7/9/3/18/5) mimicking the composition of the liver^35^ were prepared. The lipid composition was chosen according to previous publications reporting the composition of membranes isolated from brain^33,34^ and liver^35^. The lipid composition of brain endothelial cells is unique, with an high amount of sphingomyelin (SM) and a lower content of phosphatidylcholine (PC) compared with other endothelial cells^33^. The relative abundance of Chol and SM, alters phospholipid packing within the membranes and phospholipid-cholesterol interactions. In particular, cholesterol interacts with sphingomylin forming rafts which influence phospholipid packing, membrane fluidity and obviously any translocation mechanism. The emission spectra of LUVs both mimicking the BBB and the liver clearly indicate the presence of ordered phases at 37 and at 25 °C; the reproducibility of the spectra after 24 h further supported that LUVs were stable and not leaky in the condition used for the experiments. The GP parameter allowed quantifying the effect of the peptide (Table 1). The fluidity of the membranes at 37 °C in the presence of gH625 was not modified significantly. In the case of BBB mimicking liposomes we observe a slight decrease of the GP parameter, indicating a shift towards more fluid membranes; on the contrary for liver mimicking liposomes we observe a slight increase of the GP value.

**Table 1:** Membrane fluidity evaluation exploiting the generalized polarization (GP) value.

	No peptide 25 °C	No peptide 37 °C	+gH625 37 °C
Liver	0.44	0.40	0.49
BBB	0.20	0.19	0.15

### Ability of gH625 to induce lipid mixing

To investigate the fusogenicity of gH625 in LUVs mimicking the composition of the BBB and of the liver, we prepared LUVs composed of PI/DOPE/DPPC/PS/Chol/SM (4/23/30/8/20/15), for the BBB and of DSPC/POPE/PI/PS/lysoPC/SM/DOPG (37/21/7/9/3/18/5), for the liver.

**Figure 4:**
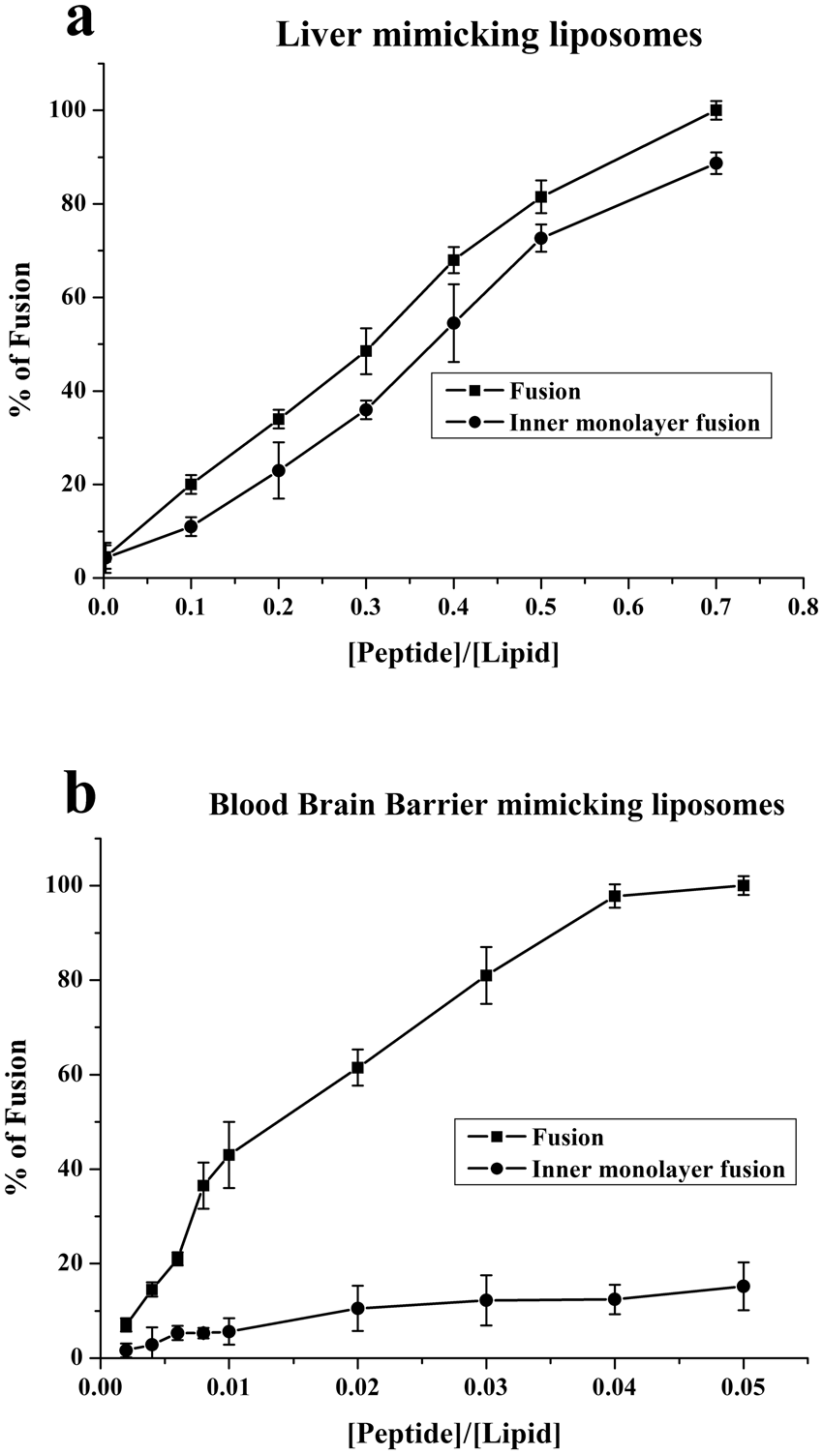
Fusion and inner monolayer promoted by gH625 in liver (panel a) and blood brain barrier (**b**) mimicking liposomes.

Increasing amounts of gH625 were added to a population of LUVs labelled with NBD (donor) and Rho (acceptor) mixed with a population of unlabelled LUVs. Fusion determines a dequenching of the donor fluorescence as a result of dilution of fluorescent vesicles. The dependence of the extent of lipid mixing on the peptide to lipid molar ratio (P/L) was analyzed and reported in Fig. 4.

Interestingly, we observed a different behavior which depended on the lipid composition of LUVs. The graphs clearly show that gH625 has higher fusogenic ability in LUVs mimicking the composition of the BBB. At a peptide/lipid ratio 10 times lower compared to the liver (maximum P/L = 0.05 in BBB; maximum P/L = 0.7 in liver) we observe complete fusion of vesicles. It is likely that the cholesterol is involved in the higher fusogenic ability of gH625 in LUVs mimicking the BBB, as we previously reported^15,49^.

In the inner monolayer assay, the fluorescence from the vesicle membranes’ outer monolayer is eliminated by the addition of an aqueous reducing agent; while in the lipid mixing experiment we measured both hemi-fusion and complete fusion, in this experiment we determined only the extent of lipid mixing between the inner monolayers. Figure 4 reports the results obtained for liposomes mimicking the BBB and the liver and shows that while for LUVs mimicking the BBB we observe approximately 20% of inner monolayer fusion at a P/L ratio corresponding to 100% of fusion, for the liver LUVs we have 100% of inner monolayer fusion at a peptide/lipid ratio where we observe also 100% of lipid mixing. Thus, in liver mimicking liposomes we observe a complete fusion which includes also the inner monolayers and could be visualized as a complete denaturation of liposomes; this takes place at high P/L ratios. For the BBB mimicking liposomes, we observe high fusion ability at very low P/L ratios and very low inner monolayer fusion in this condition.

The ANTS/DPX assay is useful to determine eventual leakage of the liposomes; in both tested conditions gH625 did not present any leakage (data not shown).

We previously reported that this set of experiments can be considered as a qualitative indicator of translocation or bilayer perturbation bearing great significance for discriminating different mechanisms of interaction. We also support the view that vesicle fusion events non complemented by leakage of the aqueous contents of the vesicle may support promising applications for drug delivery; while fusion events also accompanied by leakage are typical of antimicrobial peptides (AMPs)^15,50^.

### Tryptophan fluorescence emission analysis

**Figure 5:**
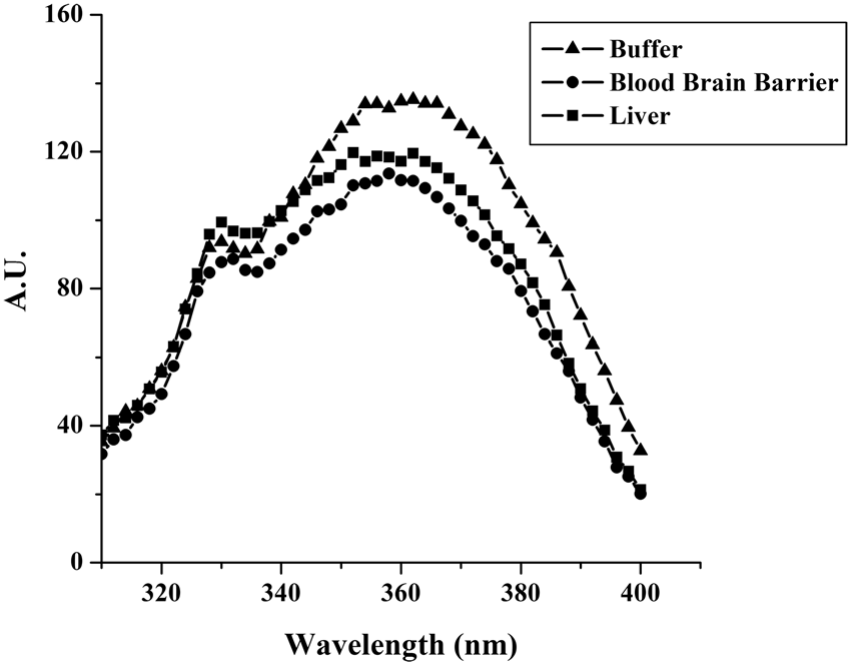
Tryptophan fluorescence emission analysis of gH625 (4 μM) in buffer and in LUVs mimicking liver and BBB.

The presence of a tryptophan residue in the middle of the sequence of gH625, allowed the evaluation of the degree of peptide penetration into liposomes of different composition. In particular, we compared the fluorescence emission spectra in vesicles mimicking the BBB and the liver with that in buffer. The quantum yield of aromatic residues of a peptide or protein normally changes when the amino acid is located in a more hydrophobic environment such as a phospholipid membrane, with an increase of the intensity of the fluorescence emission and a shift of the maximum toward shorter wavelengths (blue shift). Changes in the spectral properties were observed for both vesicles, suggesting that the single tryptophan residue of gH625 moves to a less polar environment upon interaction with lipids. Emission intensity was enhanced and a slight shift of the maxima towards lower wavelength was observed (Fig. 5). Blue shifts of this magnitude are typical of amphiphilic aromatic peptides interacting with membrane bilayers and are correlated to the aromatic moiety becoming partially immersed in the membrane. The observed blue shifts suggest that the peptide is capable of penetrating both lipid bilayers.

**Figure 6:**
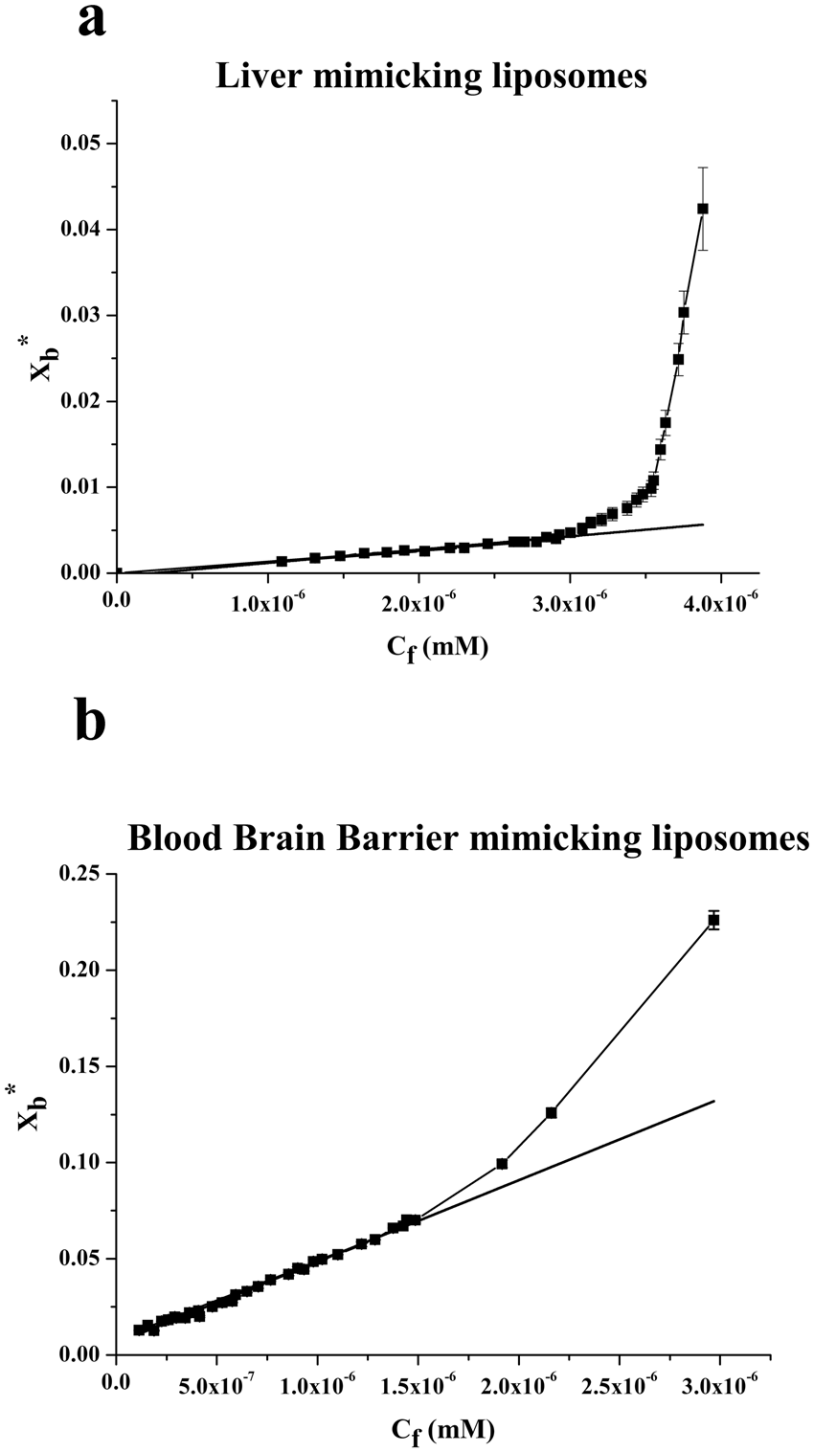
Binding isotherms in LUVs mimicking liver and BBB obtained plotting Xb* versus Cf for gH625 the peptide concentration for the spectra is 4 μM and the lipid/peptide ratio is 200.

The change in fluorescence for the tryptophan binding to phospholipids was exploited to obtain binding isotherms for gH625 in the two different membrane mimetic environment and thus calculation of partition coefficients. The gH625 concentration is low enough to cause minimal aggregation in the aqueous phase and does not disrupt the bilayer structure. In order to determine the surface partition coefficients in LUVs mimicking the BBB and the liver, the fluorescence intensities were converted to moles of bound peptide per moles of lipid and plotted as a function of the free peptide concentration (C_f_) as described in the experimental section. Partition coefficients depend on the concentration of lipid accessible to peptide, thus the curves obtained by plotting X_b_^*^ (the molar ratio of bound peptide per 60% of the total lipid) vs C_f_ (the equilibrium concentration of free peptide in the solution) are referred to as the conventional binding isotherms and are reported in Fig. 6. The shape of the binding isotherm was analyzed to get some insights into the organization of the peptide within the membrane; in fact, a straight line indicates a simple adhesion process, while a non-linear graph indicates a cooperative association of the peptide on the surface typical of peptides that self-associate at membrane surfaces upon partitioning. The shape of the isotherms obtained in both experimental conditions reflect a process whereby peptides first incorporate into the membrane and then aggregate there within; the opposite course of the isotherms would have been obtained in case of aggregation only in the water but not in the bilayer phase. The bound peptide (X_b_^*^) slowly increases until a critical concentration is reached, where massive internal aggregation apparently starts to develop. The surface partition coefficients K_p_ were estimated by extrapolating the initial slopes of the curves to C_f_ values of zero. The K_p_ values are shown in Table 2. In particular, the K_p_ value obtained for the BBB is 5.7 10^4^, indicating that the tryptophan in LUVs mimicking the BBB is able to interact significantly with the bilayer and that most of the peptide is located inside the liposomes; the K_p_ value for LUVs mimicking the liver is 5.7 10^3^, indicating that also in this case the tryptophan is located inside the liposomes and is stably inserted but with a lower affinity.

**Table 2:** Partition coefficients (Kp) and Stern-Volmer (Ksv) quenching constants.

	Buffer	BBB	Liver
K_p_	—	(4.24 ± 0.06) 10^4^	(1.55 ± 0.08) 10^3^
K_sv_(M^−1^)	10.21 ± 0.15	2.05 ± 0.08	3.04 ± 0.06

### Quenching of tryptophan by acrylamide

**Figure 7:**
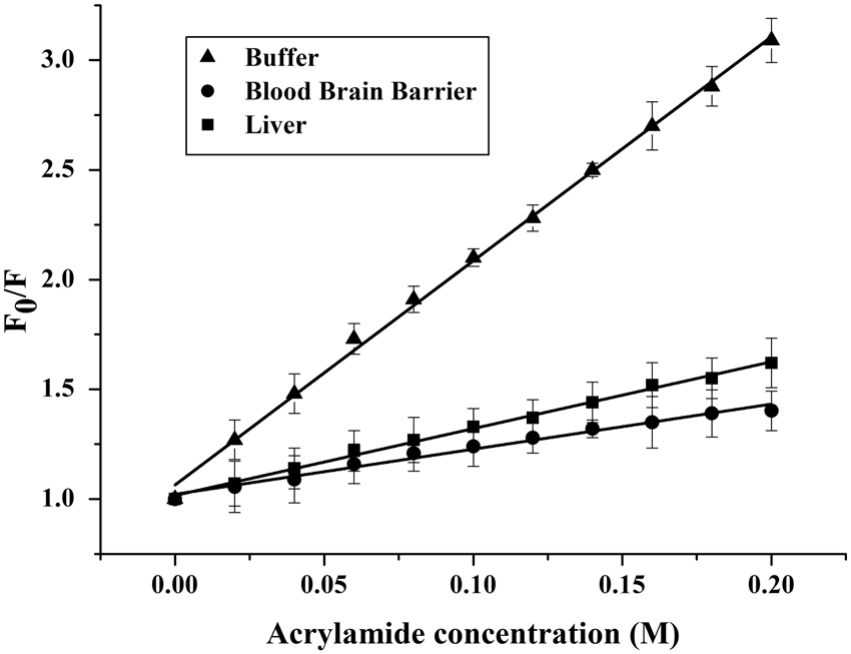
Quenching of tryptophan by acrylamide. Stern-Volmer plots of acrylamide quenching of gH625in buffer, in LUVs mimicking liver and blood brain barrier.

The observed changes in the tryptophan emission upon binding to lipid vesicles indicate gH625 insertion into the hydrophobic region of the bilayers. We determined the accessibility of the tryptophan residues of membrane bound peptides towards acrylamide, a neutral, water-soluble quencher, which is unable to penetrate into the hydrophobic core of the lipid bilayer. In fact, tryptophan residues more deeply buried inside the membrane are less strongly quenched. Stern-Volmer plots for the quenching of tryptophan by acrylamide are shown in Fig. 7.

In the presence of both types of LUVs, a great decrease in fluorescence intensity was evident, thus revealing that the tryptophan is less accessible to the quencher. In fact, the values for K_sv_ were lower (Table 2) in LUVs, suggesting that the tryptophan is more buried in the bilayers. Results clearly show that the tryptophan is well-inserted inside the bilayer in both situations but we observe a deeper insertion for the LUVs mimicking the BBB.

### Secondary structure

**Figure 8:**
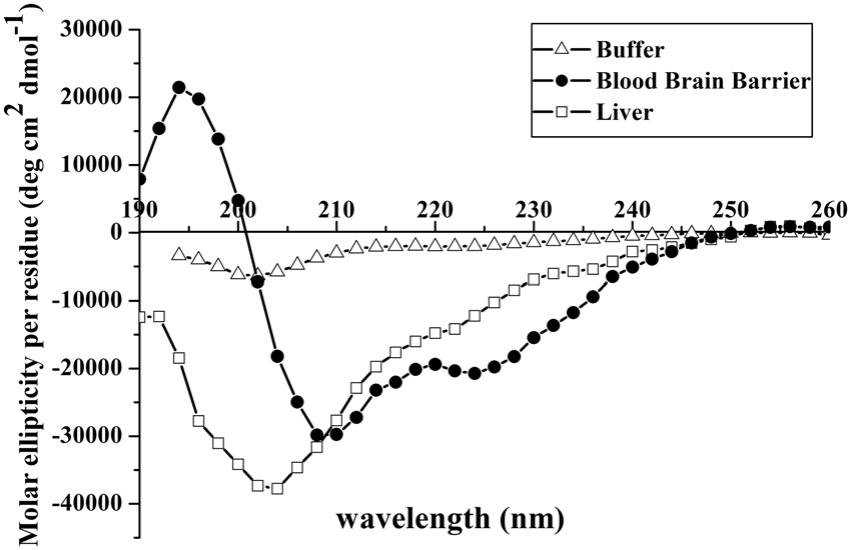
Circular dichroism spectra of gH625 in LUVs mimicking liver and blood brain barrier. Peptide concentration used is 8 μM, lipid concentration used is 100 μM and peptide/lipid ratio is 0.08.

Since the structural conformation has been shown to relate to internalization, the secondary structure was determined by CD spectroscopy as measured in water and in liposomes of the two different compositions to reveal changes of the secondary structure with respect to different experimental conditions (Fig. 8).

gH625 exhibits a mainly random coil structure in aqueous solution. In the presence of SUVs mimicking the BBB and the liver, the peptide assumes a helical conformation with two negative bands at about 208 and 222 nm. The calculations of helix content according to Chakrabartty *et al*.^44^ gives a 58% for gH625 in SUVs mimicking the BBB and 40% in SUVs mimicking the liver. Notwithstanding the percentages of helix calculated, the visual analysis of the spectra clearly indicates a more significant helical structure for BBB.

To determine whether we were also observing oligomerization processes in the experimental condition used for our assays we calculated the ratio of the ellipticities at 222 and 208 nm, which helps in discriminating between monomeric and oligomeric states of helices^45^. In fact, when the ratio θ_222_/θ_208_ is lower than 0.8, the peptide is essentially in its monomeric state, and when the ratio exceeds the value of 1.0, it is in its oligomeric state. The data reveal that in both conditions, gH625 adopts a α-helical conformation with the monomer/oligomer equilibrium shifted toward the monomeric state with a ratio θ_222_/θ_208_ of approximately 0.68 in SUVs mimicking the BBB and 0.45 in SUVs mimicking the liver. Moreover, as expected we did not observe any effect on the spectra after centrifugation to remove eventually precipitated peptides from solution.

The change of conformation from random to helical is a key feature and the driving force of peptides able to enter the bilayer. Thus, the obtained results clearly indicate that gH625 is able to change from a random coil to a helical structure in vesicles mimicking the BBB, while the conformational change is much less evident in vesicles mimicking the liver.

## Discussion

We previously found that gH625 can facilitate brain delivery^24^ although the mechanism by which gH625 mediates the crossing of the BBB and liver barriers remains unclear. We focussed on the fact that probably the mechanisms promoting uptake is different. While much is known on how gH625 is crossing the membranes of eukaryotic cells in general less is known specifically for liver and BBB.

Our previous findings showed that gH625 was able to reach the brain after intravenous injection, despite macrophages of liver (Kupffer cells), seem to sequestrate the peptide from circulation^24^. We also previously reported that gH625 is worth for further development as a suitable device to enhance drug delivery beyond the BBB since it presents a high serum stability (after 3.5 h of incubation we could find still a significant amount of intact peptide) and the percentage of uptake in human neuroblastoma and glioblastoma astrocytoma is around 80–90%^24^. Nonetheless, we did not perform a comparative evaluation of the BBB uptake with that of the liver. Any such information is crucial for the eventual application of gH625.

Thus, this work enlarges our knowledge on the biodistribution of gH625 in an animal model showing that *in vivo* administration of gH625 permits to reach the BBB, although part of the peptide is sequestered by liver. In particular, our data clearly show that gH625 reaches the BBB and crosses it; this is the first *in vivo* quantification of gH625 crossing the BBB. Interestingly, we demonstrated that gH625 is able to reach the brain at lower doses injected with higher percentages of delivered peptide compared to the liver. These data further show that, despite the liver is able to withdraw gH625, and although we cannot state whether the reason of the liver accumulation is macrophage activation with consequent gH625 removal or endothelial generalized uptake, the administration of lower amounts of peptide should be more effective for brain delivery.

The comprehension of the mechanism of interaction of gH625 with model membranes mimicking the BBB and the liver allows obtaining key information, which could be useful for the design of novel drug delivery vectors. It is important to note that CPPs present numerous similarities with AMPs in charge, structure etc. and it is key to understand how these parameters can influence their interaction with the membrane and affect their translocation ability and cytotoxicity^50,51^. In particular, we observed that gH625 presents a greater fusion ability for membranes mimicking the BBB while lower fusion ability for liver mimicking membranes; moreover, from the inner monolayer experiment, we note that in condition where we observe complete lipid mixing we do not observe significant inner monolayer mixing while for liver membranes in condition with complete lipid mixing we have also significant inner monolayer mixing. This result clearly indicates that we have hemi-fusion only (outer membrane fusion) for BBB membranes and complete fusion for liver. The results obtained can also be correlated to the presence of a high percentage of cholesterol which is typical of lipid rafts. It is well known that BBB endothelial membranes are characterized by lipid rafts and we also previously reported the higher ability of gH625 to interact with membranes containing a high amount of cholesterol^26,27,52^. Moreover, we do not observe leakage in both conditions, which is key for eventual applications in drug delivery and discriminates CPPs from antimicrobial peptides^53–55^. Furthermore, these experiments hold great importance as qualitative indicators of translocation or bilayer perturbation bearing great significance for penetration across bilayers.

The analysis of the fluorescence spectra allowed the determination of affinity constants for the BBB and the liver membranes and further supports our results indicating a stronger interaction with the BBB and a deeper insertion of the tryptophan inside liposomes mimicking the BBB.

Secondary structure plays a major role in the uptake; thus, a key experiment is the determination of the conformation in different environments. A conformational change is key for modulation of peptide/lipid interactions and in particular, structural plasticity (the ability to change conformation according to the environment) has a crucial role in penetration across the membranes. In our study, we clearly observe a different structure of gH625 when moving from an aqueous solution to membranes. When in aqueous solution, the peptide has a random coil structure, but in the membranes it adopts a helical conformation with a higher helical content in membranes mimicking the BBB. Changes in the secondary structures are key for the translocation across membranes and the change in secondary structure represents the driving force for the penetration. This result is consistent with previous finding showing that pVEC adopts a β-sheet structure and TP10 becomes α-helical^9,56,57^.

## Conclusions

One of the major challenges of current research is drug delivery for Central Nervous System (CNS) associated pathologies such as Alzheimer, Parkinson, brain injury, cancer^58,59^. Intravenous administration may represent a relatively non-invasive route of drug delivery; however, the BBB is a key obstacle for both small and large molecules^58,60^. Improvement of brain delivery and reduction of systemic exposure of drugs are key points which may enable delivery of therapeutics from the bloodstream to the brain^61,62^. To better understand and ameliorate the physicochemical properties of peptide drug delivery vectors to improve accumulation on target tissue, biodistribution studies are of great relevance. As a general rule, organs with high blood flow and rich in reticulo-endothelial system such as liver, could accumulate cell penetrating peptides^63,64^. Biodistribution analysis on other CPPs showed that the distribution among the different tissue depends on the peptide but all present a high liver distribution^9,13^.

**Figure 9:**
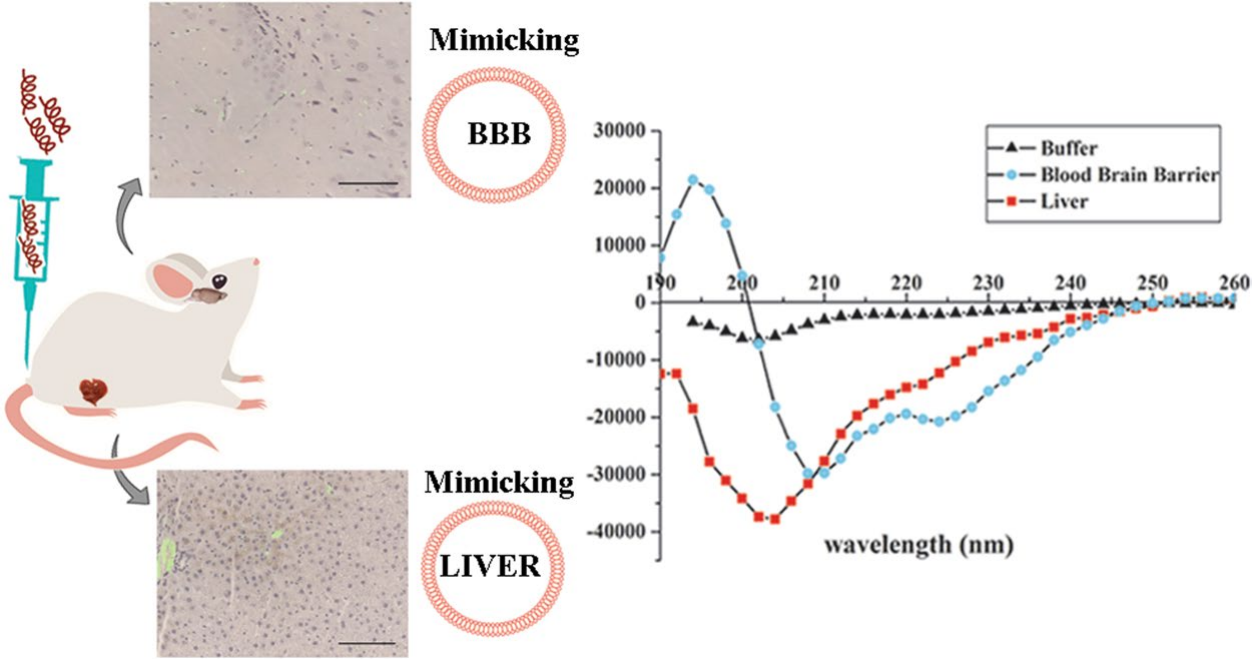
Graphical abstract. The mouse drawing was created by Freepik.

Overall, our results indicate that the physicochemical properties such as the secondary structure in aqueous and membrane environments influence the cellular uptake mechanism and thus the biological properties (Fig. 9). In fact, despite the liver withdrawal of gH625, this peptide has the ability to reach and cross the BBB and this feature is ameliorated by lowering its concentration in blood. In conclusion, BBB peptide shuttles such as gH625 are a valid opportunity to enhance brain delivery of therapeutics and biophysical studies may be useful in improving their advantages compared to other drug delivery tools.

## Electronic supplementary material

Supplementary information
