# Supplementary material for: Enhanced uptake of gH625 by blood brain barrier compared to liver in vivo: characterization of the mechanism by an in vitro model and implications for delivery
Source: Sci Rep. 2018 Sep 14;8:13836. doi: 10.1038/s41598-018-32095-w (PMC6138628; doi:10.1038/s41598-018-32095-w)
Supplement: Supplementary file 1 — Supplementary information [file 41598_2018_32095_MOESM1_ESM.pdf]

Enhanced uptake of gH625 by blood brain barrier compared to liver *in vivo*: characterization of the mechanism by an *in vitro* model and implications for delivery.

Annarita Falanga,[1,2] Giuseppina Iachetta,[3] Lucia Lombardi,[1] Emiliana Perillo,[1] Assunta Lombardi,[3] Giancarlo Morelli,[1,2] Salvatore Valiante,[3,4] Stefania Galdiero[1,2]*

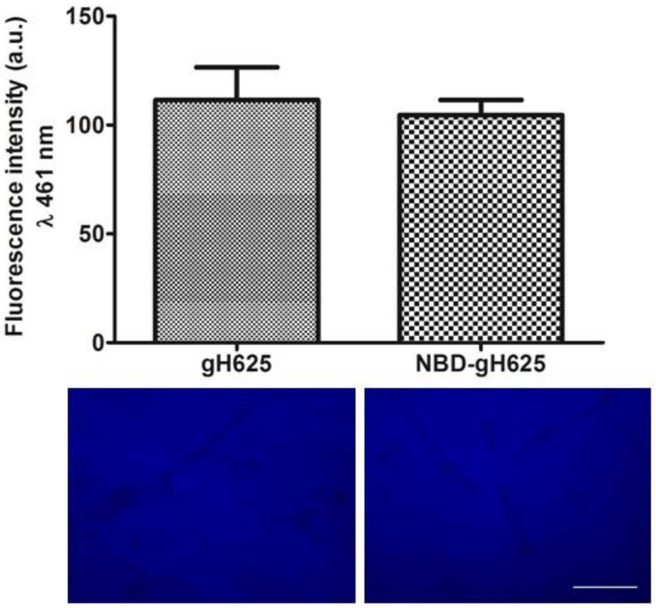

Figure S1. Evaluation of the fluorescence signal on ex vivo brain samples at 461 nm. Images show no difference in signal between gH625 and NBD-gH625 samples. Scale bar 50 μm.
